# Supplementary material for: Seed coat-derived brassinosteroid signaling regulates endosperm development
Source: Nat Commun. 2024 Oct 29;15:9352. doi: 10.1038/s41467-024-53671-x (PMC11522626; doi:10.1038/s41467-024-53671-x)
Supplement: Supplementary file 1 — Supplementary Information [file 41467_2024_53671_MOESM1_ESM.pdf]

**Seed coat-derived brassinosteroids non-cell autonomously contribute to endosperm development**

# Supplementary materials

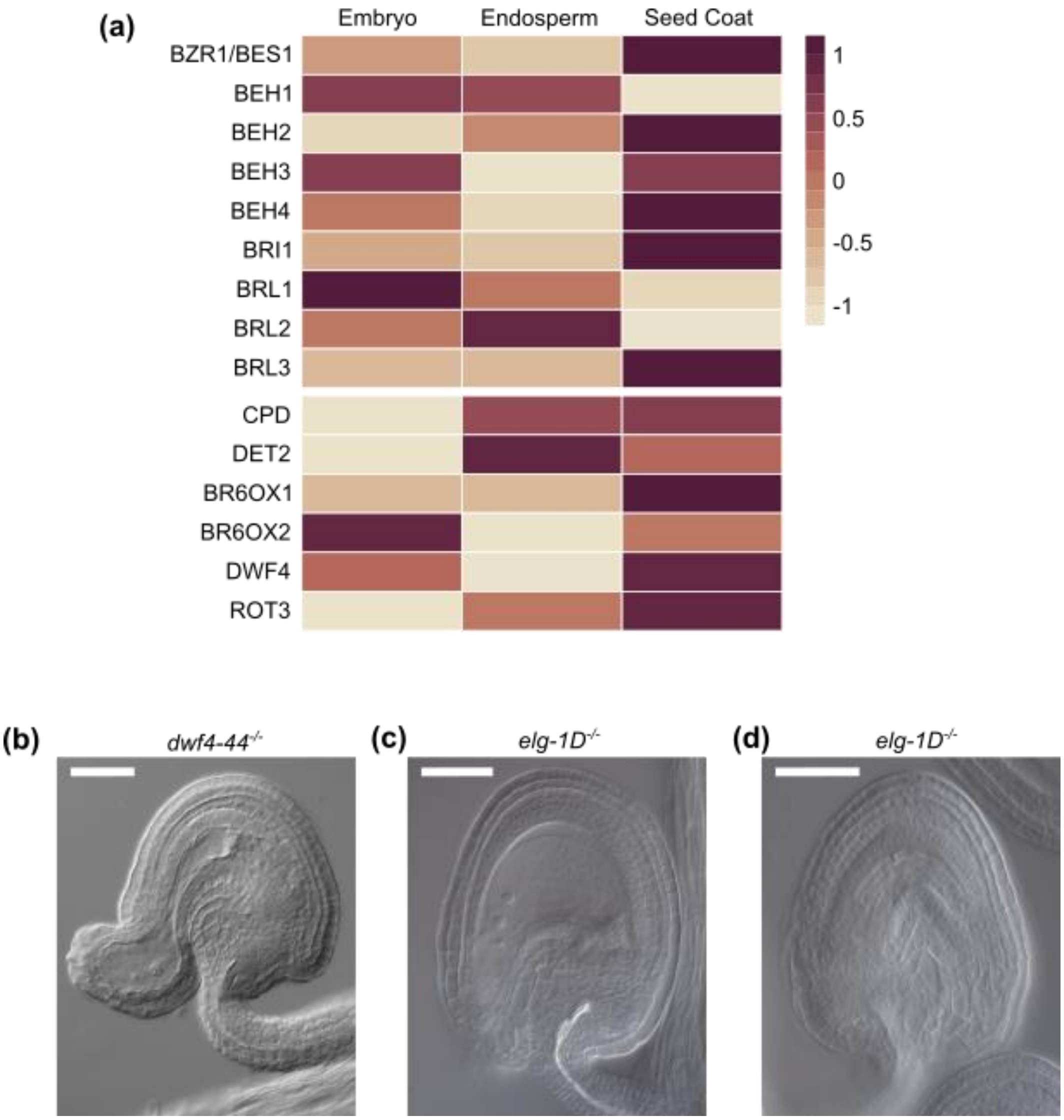


**Supplementary Figure 1**. **BR are putative regulators of seed development**. (a) Relative expression of BR signalling and biosynthetic genes in the embryo, endosperm and seed coat at 3 DAP. Z-score normalization was performed to center the mean and set the distribution of values to a SD of 1. (b-d) Examples of malformed ovules of mutants impaired in BR function. *dwf4-44*$^{-/-}$ (b) ovule with underdeveloped outer integuments and *elg-1D*$^{-/-}$ ovules with unfused polar nuclei (c) and persistent nucellus (d) at 5 days after emasculation (DAE). Scale bars, 50 µm. These experiments were repeated three times with similar results.

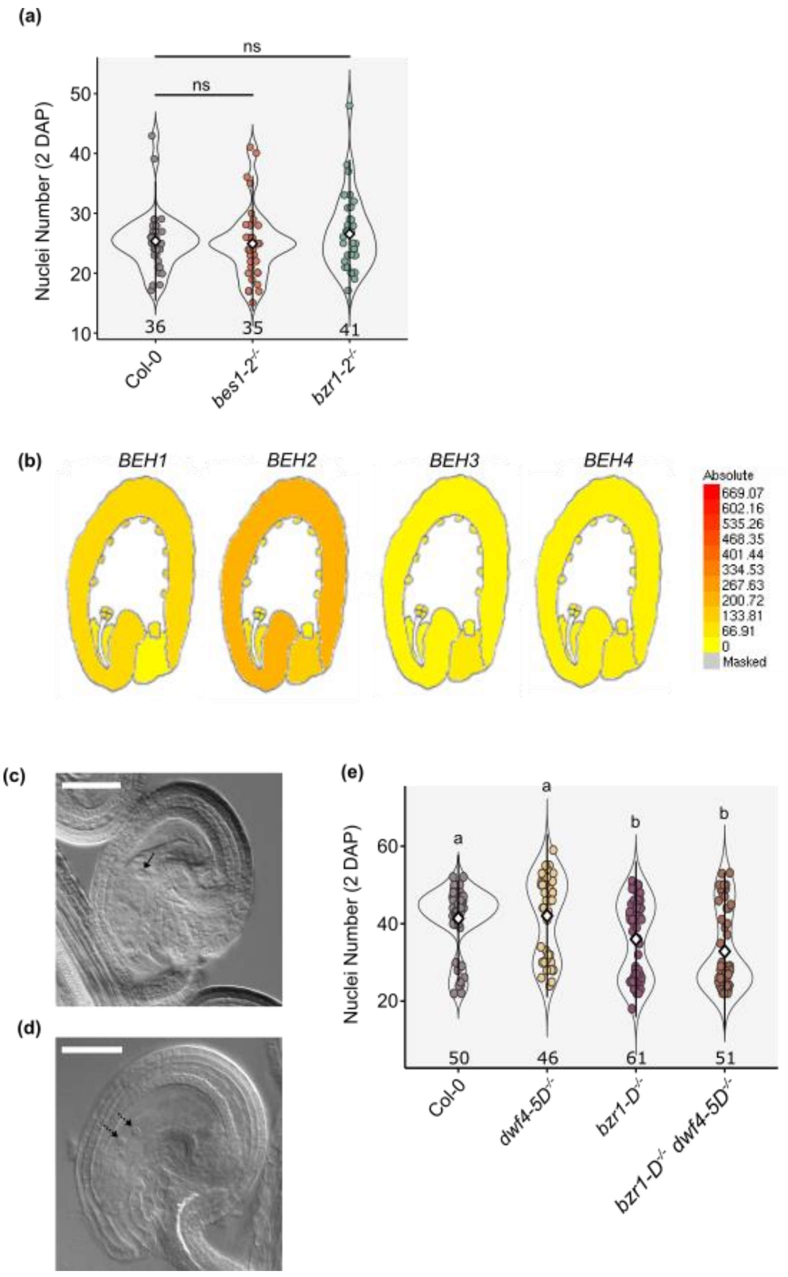


**Supplementary Figure 2**. **BZR1/BES1 family of TFs and endosperm development**. (a) Endosperm nuclei number at 2 days after pollination (DAP) for BR mutants and WT. Numbers at the bottom indicate number of seeds analysed. Significance of differences were determined by one-way ANOVA. (b) expression map of *BES1/BZR1* homologs *BEH1*, *BEH2* , *BEH3* and *BEH4* (taken from eFP Browser). (c-d) Photos of a WT (c) and a *bzr1-D* ovule (d), showing unfused polar nuclei at 5 DAE (arrows). Scale bars, 50 µm. These experiments were repeated three times with similar results. (e) Endosperm nuclei number for WT, *dwf4-5D*$^{-/-}$, *bzr1-D*$^{-/-}$ and the double mutant *bzr1-D*$^{-/-}$ dwf4-5D$^{-/-}$ at 2 DAP. Numbers at the bottom indicate number of seeds analysed. Error bars represent standard deviation. Letters indicate significance of differences determined by Tukey's HSD test after one-way ANOVA test.

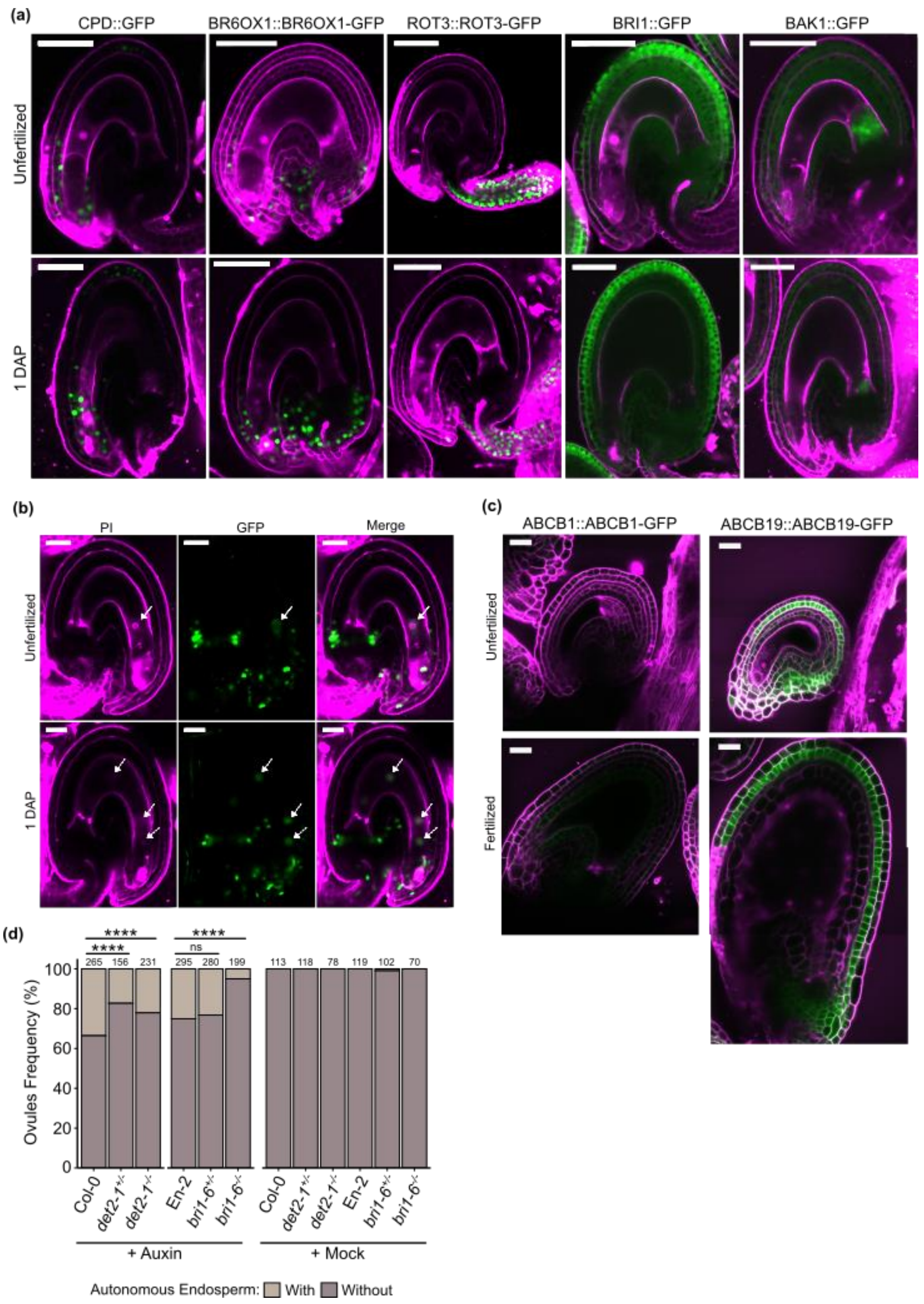


**Supplementary Figure 3. BR effect on endosperm development is sporophytic**. (a) Expression pattern of *CPD::GFP, BR6OX1::BR6OX1-GFP, ROT3::ROT3-GFP, BRI1::GFP* and *BAK1::GFP* in unfertilized ovules (upper row) and seeds at 1 DAP (bottom row). Ovules and seeds were stained with propidium iodide (in magenta). Scale bars, 50 µm. These experiments were repeated three times with similar results. (b) Representative image of an ovule (upper panels) and seed at 1 DAP (lower panels) showing *BR6OX2::BR6OX2-GFP* expression in the central cell and endosperm, respectively. Solid arrows indicate central cell nucleus and dotted arrows point to endosperm nuclei. Ovules and seeds were stained with propidium iodide (in magenta). Scale

bars, 25 µm. This experiment was repeated twice with similar results. (c) Expression pattern of *ABCB1::ABCB1:GFP* and of *ABCB19::ABCB19:GFP* in unfertilized ovules and in fertilized seeds. Scale bars, 25 µm. Ovules and seeds were stained with propidium iodide (in magenta). These experiments were repeated three times with similar results. (d) Percentage of *det2-1*$^{+/-}$, *bri1-6*$^{+/-}$, and respective WTs, ovules producing autonomous endosperm development at 3 days after exogenous auxin application. The values on top of each bar indicate the number of ovules analysed. Significance of differences were determined by a one-way chi squared test **** $p<0.0001$. Exact p-values are found in the Source Data file.

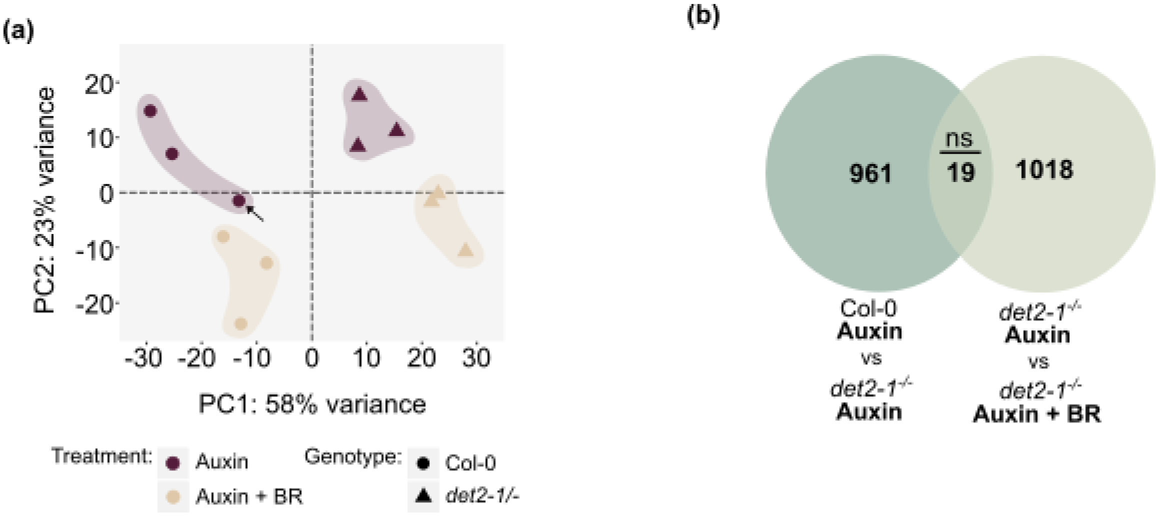


**Supplementary Figure 4. RNAseq analysis of BR treatments.** (a) Principal component analysis of the samples used for the RNAseq experiment, showing PC1 and PC2. The sample indicated with an arrow was removed from the final analysis, since it did not cluster with the other two replicates. (b) Venn diagram showing overlapping genes that are downregulated in WT versus *det2-1*$^{-/-}$ ovules treated with auxin and upregulated in *det2-1*$^{-/-}$ ovules treated with auxin and BR versus auxin. The overlap between datasets was not statistically significant as determined by a one-way hypergeometric test.

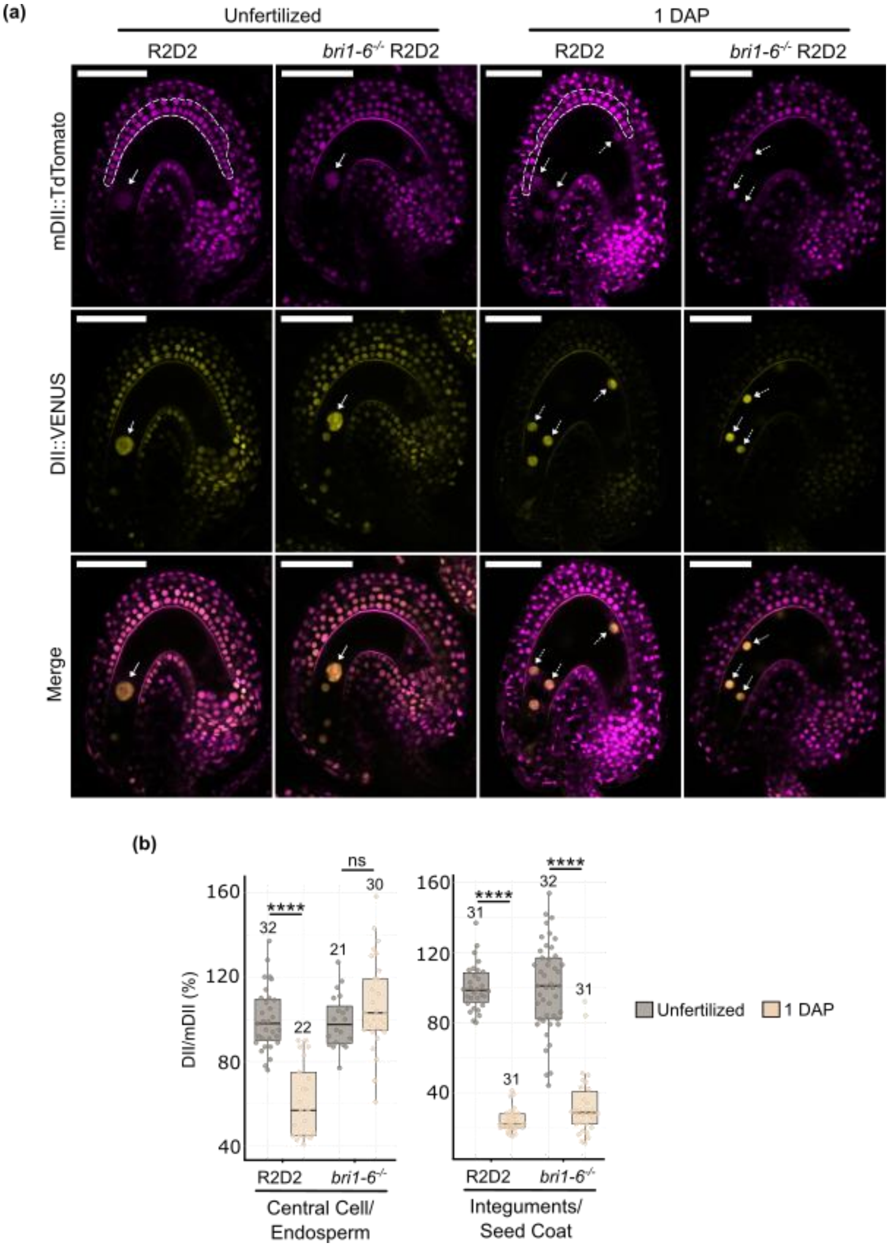


**Supplementary Figure 5. Auxin activity is reduced in *bri1-6* endosperm.** (a) Representative microscopy images of WT and *bri1-6*$^{-/-}$ unfertilized ovules and seeds at 1 DAP expressing the R2D2 auxin sensor. Solid arrows indicate the central cell nucleus and dotted arrows point to endosperm nuclei. Dashed lines surround the nuclei of the integuments and seed coat layers used for quantification. Scale bars, 50 µm. These experiments were repeated twice with similar results. (b) Quantification of DII-Venus/mDII-ntdTomato signal in R2D2 and *bri1-6*$^{-/-}$ seeds before and after fertilization. The values on top of each bar indicate the number of ovules/seeds analysed. Error bars represent standard deviation. Significance of difference was determined by two-tailed distribution Student test. **** $p<0.0001$. Exact p-values are found in the Source Data file.

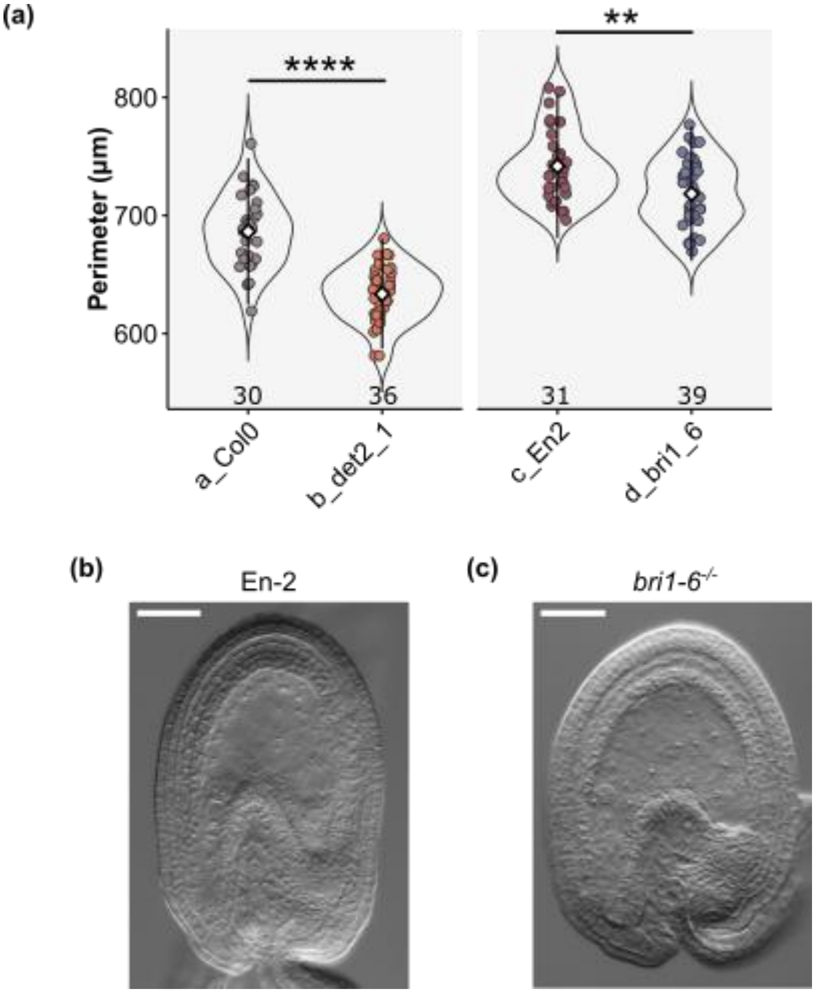


**Supplementary Figure 6. *det2-1* and *bri1-6* seeds are smaller than the WT**. (a) Perimeter of *det2-1*$^{-/-}$ and *bri1-6*$^{-/-}$ seeds comparatively to the respective WT at 2 DAP. Numbers at the bottom indicate number of seeds analysed. Significance of differences were determined by one-way ANOVA. **** p<0.0001, *** p<0.001, ** p<0.01 and * p<0.05. Exact p-values are found in the Source Data files. Error bars represent standard deviation. (b-c) Representative seed of En-2 (b) and *bri1-6*$^{-/-}$ (c) at 2 DAP. Scale bars, 50 µm. These experiments were repeated five times with similar results.

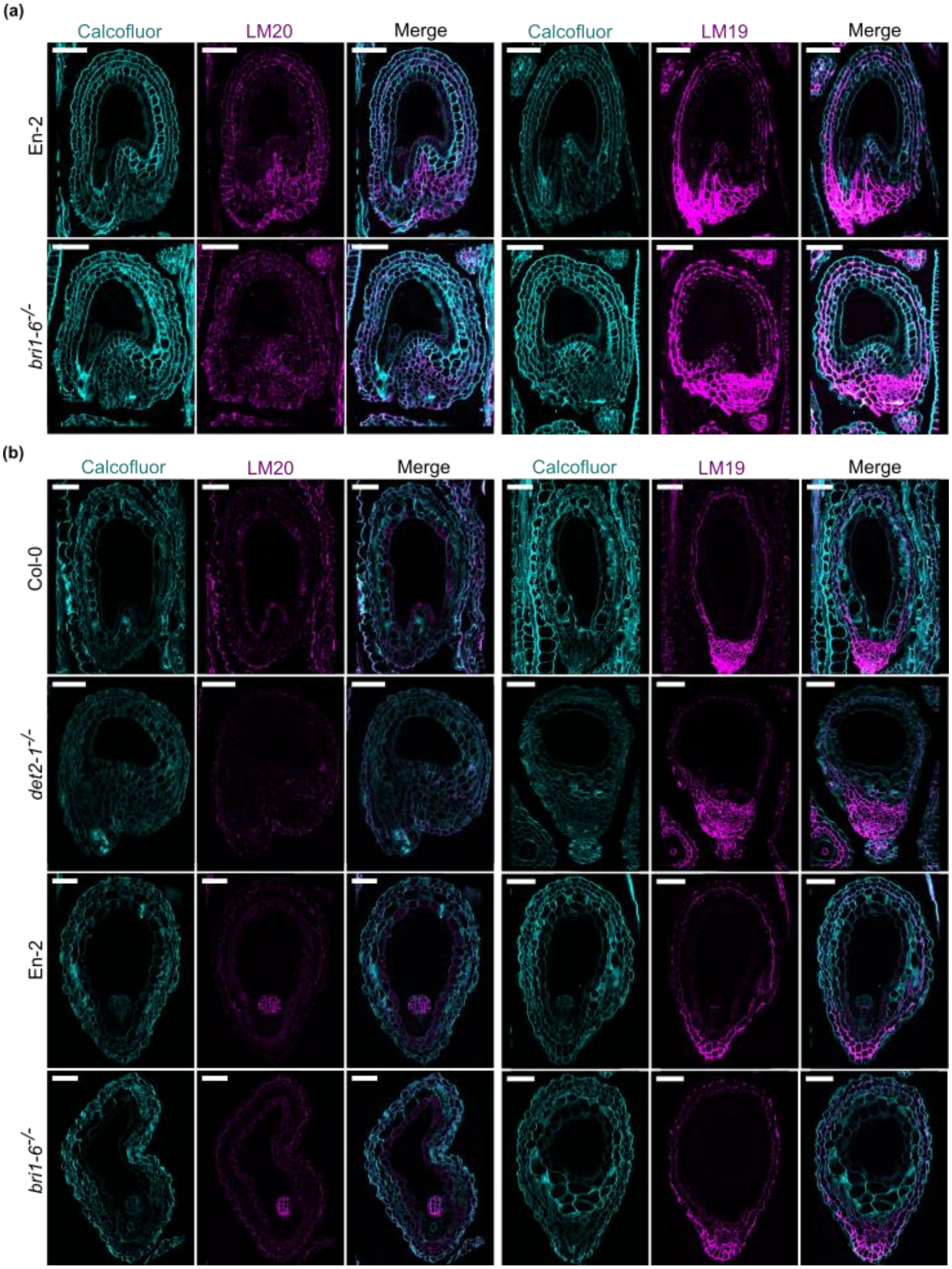


**Supplementary Figure 7. *det2-1* and *bri1-6* seed coats have altered cell wall compositions.** (a) Labelling of cell wall components in WT (upper row) and *bri1-6*$^{-/-}$ (lower row) at 2 DAP. Cyan is calcofluor white staining and purple is immunolabelling with either LM19 or LM20 antibodies. (b) Same as for (a), but for Col-0, *det2-1*$^{-/-}$, En-2 and *bri1*$^{-/-}$ at 4 DAP. Scale bars, 50 μm. These experiments were repeated twice with similar results.

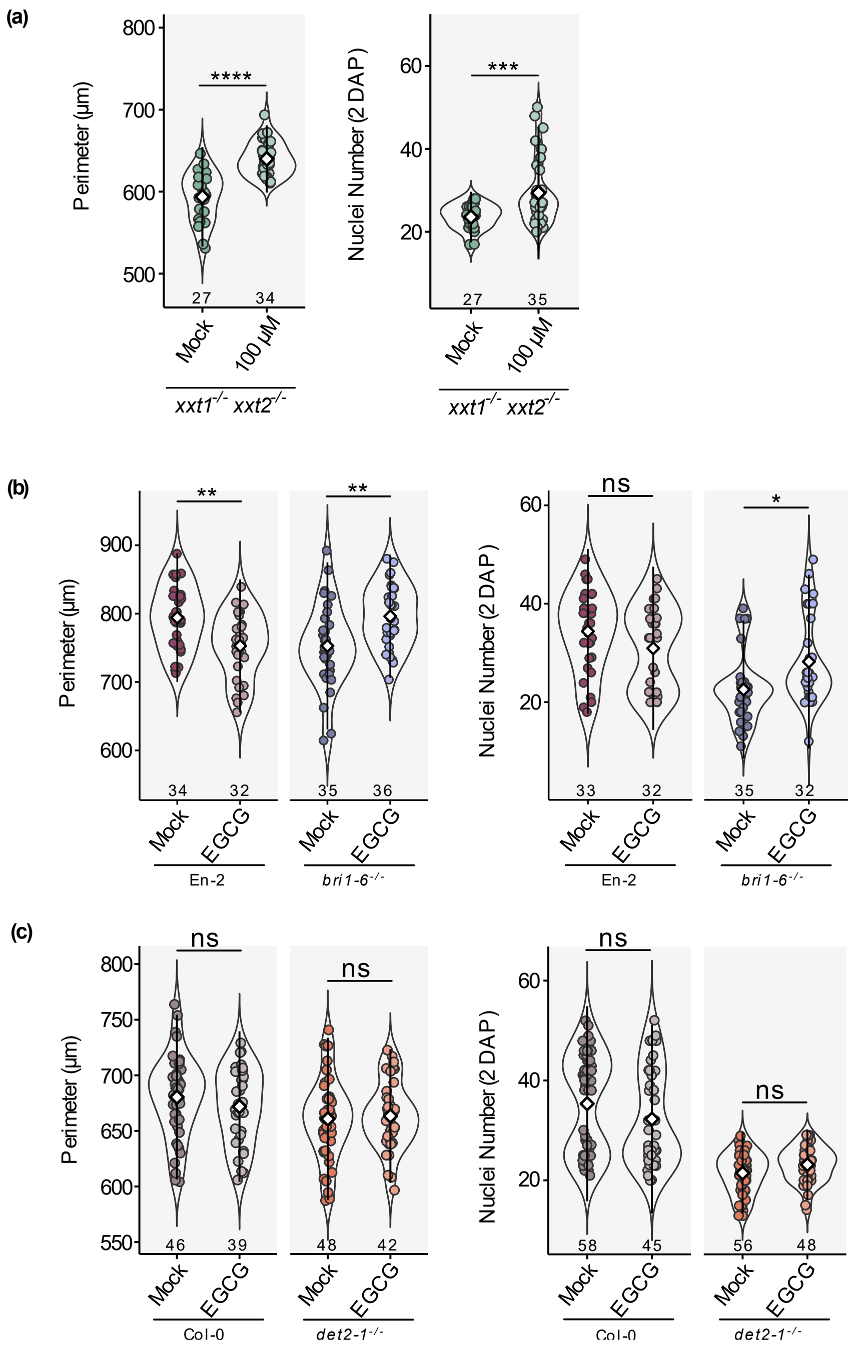


**Supplementary Figure 8. Smaller seed sizes correlate with less proliferative endosperms**. (a) Seed perimeter and endosperm nuclei number at 2 DAP in *xxt1*$^{-/-}$ *xxt2*$^{-/-}$ treated with 100 µM epi-BL. and respective WT at 3 DAT. (b-c) Seed perimeter (left) and endosperm nuclei number (right) of mock and EGCG-treated En-2 and *bri1-6*$^{-/-}$ (b) and Col-0 and *det2-1*$^{-/-}$ (c). Significance of differences was determined by one-way ANOVA. *** p<0.0001, *** p<0.001, ** p<0.01 and * p<0.05. Exact p-values can be found in the Source Data files. Error bars represent standard deviation. Numbers on the bottom indicate the number of seeds analysed.

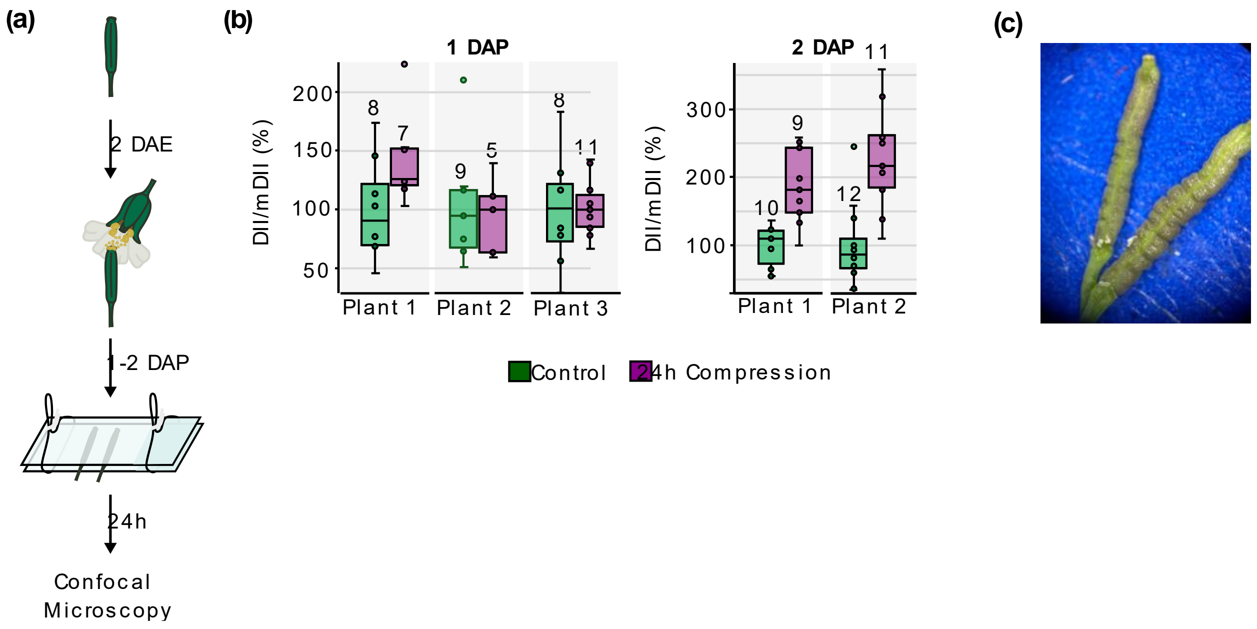


**Supplementary Figure 9. Seeds under physical pressure show reduced auxin activity in the endosperm.** (a) Experimental setup for inducing physical pressure in developing Arabidopsis fruits. (b) Quantification of DII:VENUS/mDII:tdTomato in endosperm nuclei of seeds at 1 and 2 DAP. The numbers indicate the amount of seeds analysed. Error bars indicate standard deviation. Significance of difference was determined by two-tailed distribution Student test. *** p<0.001. Exact p-values are found in the Source Data files (c) Photo of siliques at 1 DAP showing signs of compression. These siliques correspond to “Plant 1” at 1 DAP in (b). This experiment was performed twice with similar results.

**Supplementary Table 1. Primer sequences used for cloning**.

| Gene | Region | Sequence (5’ → 3’) * | |
|---|---|---|---|
| *BRI1* (AT4G39400) | Promoter | fw | GGGGACAAGTTTGTACAAAAAAGCAGGCTTACGAACGTCTGTGTTAGTC |
| | | rv | GGGGACCACTTTGTACAAGAAAGCTGGGTTTCTCAAGAGTTTGTGAGAGAG |
| *BAK1* (AT4G33430) | CDS | fw | GGGGACAAGTTTGTACAAAAAAGCAGGCTTCCCCACTCAAAAATAACAG |
| | | rv | GGGGACCACTTTGTACAAGAAAGCTGGGTTTTATCCTCAAGAGATTAAAAAC |
| *DET2* (AT2G38050) | Promoter | fw | GGGGACAAGTTTGTACAAAAAAGCAGGCTGACCACCCTACTCTCTCGGT |
| | | rv | GGGGACCACTTTGTACAAGAAAGCTGGGTTTTTCGGGTTATGGAATTGGGG |

* Primer adapters are underlined.
