# Supplementary material for: Seed coat-derived brassinosteroid signaling regulates endosperm development
Source: Nat Commun. 2024 Oct 29;15:9352. doi: 10.1038/s41467-024-53671-x (PMC11522626; doi:10.1038/s41467-024-53671-x)
Supplement: Supplementary file 2 — Description of Additional Supplementary Files [file 41467_2024_53671_MOESM2_ESM.pdf]

Description of Additional Supplementary Files

Supplementary Data 1. RNAseq analysis of WT and det2 autonomous seeds.
